# The Association of Serotonin Toxicity with Combination Linezolid–Serotonergic Agent Therapy: A Systematic Review and Meta-Analysis

**DOI:** 10.3390/pharmacy11060182

**Published:** 2023-11-20

**Authors:** Savanna SanFilippo, Jacques Turgeon, Veronique Michaud, Ronald G. Nahass, Luigi Brunetti

**Affiliations:** 1 Robert Wood Johnson University Hospital Somerset, Somerville, NJ 08876, USA; savannad.pharmd@gmail.com; 2 Ernest Mario School of Pharmacy, Rutgers, the State University of New Jersey, Piscataway, NJ 08854, USA; 3 Galenus Rx, Orlando, FL 32789, USA; jacques.turgeon@umontreal.ca (J.T.); v.michaud@umontreal.ca (V.M.); 4 IDCare, Hillsborough, NJ 08844, USA; rnahass@idcare.com

**Keywords:** linezolid, serotonin syndrome, serotonin toxicity, serotonin agonists, drug interaction

## Abstract

Linezolid (LZD) has a longstanding reported association with the onset of serotonin toxicity (ST), secondary to drug–drug interactions with serotoninergic agents. There have been no conclusive data supporting the incidence or contributing risk factors to date. The study evaluated the incidence of ST in patients treated with LZD and serotonergic agents concomitantly versus LZD alone. The secondary objectives included a comparison of ST incidence in patients treated with one serotonergic agent + LZD versus two or more serotonergic agents + LZD. The studies used for this meta-analysis were retrieved from PubMed, Scopus, and Google Scholar. All studies including a comparison between LZD alone and LZD + a serotonergic agent published between 1 January 2000 and 1 October 2023 and meeting the quality standards were considered for inclusion. Fourteen studies were identified, with five meeting all inclusion and exclusion criteria with no significant heterogeneity. For the analysis of LZD monotherapy vs. SA combination therapy, four studies with 6025 patients total were analyzed and yielded an odds ratio of 1.78 (CI [1.04, 3.02]; I^2^ = 49%; GRADE certainty: low). Four studies and 2501 patients were included in the analysis of one versus more than one SA with an odds ratio of 5.18 (CI [1.05, 25.49]; I^2^ = 44.87; GRADE certainty: moderate). The Newcastle–Ottawa score, visual inspection of the funnel plot, and Egger’s statistic were used to evaluate quality and heterogeneity. The Peto method was used to calculate the summary odds ratios. All analyses were performed using Comprehensive Meta-Analysis version 3.0 and R, while GRADE was used to evaluate the quality of the final recommendation. The number of concomitant serotonergic agents may play a role in the development of serotonin toxicity in patients prescribed linezolid. In patients requiring linezolid therapy and serotonergic agents, risk versus benefit analysis should pay attention to the number of interacting drugs.

## 1. Introduction

Linezolid (LZD), an oxazolidinone antibiotic indicated for use in certain Gram-positive infections, may interact with serotonergic agents leading to serotonin (5-hydroxytryptamine (5-HT)) toxicity (ST) based on case reports and the proposed mechanism of action [1,2]. To understand the proposed mechanism of interaction, it is important to also understand the mechanism of serotonin toxicity. ST itself is caused by an excess of serotonin in the central nervous system (CNS), which can be caused by the inhibition of serotonin metabolism, reuptake prevention, or increased serotonin release. The excess CNS serotonin acts on various serotonin receptors, leading to the symptoms of ST, such as rigidity and hyperthermia [1,2]. The proposed mechanism of action of the interaction between LZD and serotonergic agents leading to ST is logically related to the accumulation of excess serotonin in the CNS [1,2]. First is the nonselective inhibition of monoamine oxidase (MAO) by LZD [1,2]. In turn, this blocks the metabolism of monoamine neurotransmitters and creates an excess of serotonin in the CNS [1,2]. For patients taking serotonergic agents, this interaction is of particular concern because of the proposed additive CNS accumulation of serotonin from the administration of both serotonergic agents and LZD. Although there is no concrete evidence describing an accurate incidence rate, the consensus is that this event is rare [3,4,5,6]. 

The original clinical trials of LZD, when LZD first received approval by the Food and Drug Administration, did not find any incidence of ST [3,5,6]. However, since the approval of LZD in the year 2000, there have been many case reports involving ST and LZD given with serotonergic agents [6]. In 2011, the FDA issued a warning to avoid concomitant LZD plus serotonergic agent therapy [4]. The current FDA recommendation is to provide a 14-day washout for patients taking serotonergic agents before starting LZD [4]. As it is unpredictable when LZD may be required to treat an infection, this recommendation is difficult to implement. For example, many serotonergic agents, such as serotonergic reuptake inhibitors and other antidepressants, should be tapered slowly to avoid withdrawal symptoms. If the serotonergic agent was properly tapered and the recommended 14-day washout period followed, a patient would have not only an increased risk of recurring or worsening psychiatric symptoms but also a further delay of LZD initiation. Thus, clinicians are faced with the dilemma of choosing between the timely treatment of infection and the potential for serotonin toxicity secondary to concomitant LZD + serotonergic agent therapy. It may be challenging to weigh the risks and benefits in this scenario. On the one hand, there is insurmountable evidence for rapid antibiotic administration. However, there is not enough strong evidence regarding the incidence of ST when given serotonergic agents and LZD concomitantly. This evidence inequality makes it challenging to guide the decision in many cases. These complex considerations underscore the critical need for additional research to better inform clinical decision-making, balancing infection treatment urgency with managing potential ST risks in LZD and serotonergic agent coadministration.

Understanding the true risks of ST when LZD is prescribed with serotonergic agents will provide clinicians with the needed guidance to support treatment decisions while enhancing patient safety. Observational studies and case series have sought to identify the risk factors and incidence of ST caused by LZD plus serotonergic agent therapy. They have found little to no ST caused by LZD plus serotonergic agent administration, but, to date, no robust analyses have been performed to strengthen or confirm their findings. Despite this, the combination is still cautioned against by the FDA and others [5,6,7,8,9,10,11,12,13]. Considering the current state of evidence and regulatory caution, further comprehensive analyses are needed to definitively assess the safety profile of LZD in conjunction with serotonergic agents, providing clinicians with the necessary data for informed treatment decisions and ensuring patient well-being.

We sought to assess if there is a higher incidence of ST in the general medical population when LZD is used concomitantly with a serotonergic agent compared to LZD use alone. The primary objective of this analysis was to define the incidence of ST in patients treated with LZD alone and when prescribed concomitantly with serotonergic agents. The secondary objectives included a comparison of ST incidence in patients treated with one serotonergic agent + LZD versus two or more serotonergic agents + LZD. By examining the incidence of ST in patients treated with LZD alone compared to those receiving LZD concomitantly with serotonergic agents, this study aims to shed light on a critical safety concern, offering clinicians valuable data to navigate the complex landscape of the timely administration of LZD and safe administration of serotonergic agents. 

## 2. Materials and Methods

Our research methodology was conducted in accordance with the 2020 PRISMA Guidelines, ensuring a comprehensive and systematic approach to our meta-analysis [14]. To provide transparency and adherence to best practices, a completed PRISMA 2020 Checklist for this meta-analysis can be found in Table A1. It is worth noting that the protocol for this meta-analysis was not prospectively registered, a limitation that we openly acknowledge. In subsequent sections of this paper, we highlight its implications and provide insights into how it may have influenced our findings. 

### 2.1. Eligibility Criteria

To be included in the study, articles had to include either a comparison between LZD + serotonergic agents to LZD alone or a comparison between LZD + 1 serotonergic agent to LZD + multiple serotonergic agents. Studies could be randomized controlled trials, observational studies, or case series. Although randomized controlled trials were eligible for the study, none were found in the literature search. Moreover, each study selected was required to report key outcomes of interest, such as the rate of ST and the number of concomitant serotonergic agents. For statistical reasons, studies with zero incidence of ST in either comparator group were also excluded. Lastly, each study had to meet the quality standard of a Newcastle–Ottawa Scale (NOS) over 5.

### 2.2. Information Sources

From 1 May to 1 October 2023, we conducted our search across multiple databases; we queried PubMed, SCOPUS, and Google Scholar for relevant articles meeting the inclusion and exclusion criteria. To increase our ability to capture all relevant literature meeting the inclusion and exclusion criteria, we also reviewed the references of each included article. 

### 2.3. Search Strategy

The search was performed in all three databases using (“linezolid” AND “serotonin syndrome”), (“linezolid” AND “serotonin toxicity”), and (“linezolid” AND “serotonin agonists”) and (“linezolid” AND “serotonergic agents”). The review was restricted to studies published from 2000, when LZD was first available, until May 2023 [3]. The terms were entered into each database as listed; the filter function was used in each respective database to exclude case studies and animal studies. No language criteria were applied. After identifying relevant studies, titles and abstracts were further reviewed for inclusion and exclusion criteria. 

### 2.4. Selection Process and Data Collection Process

Two independent reviewers performed the search strategy (SS), study selection (SS and LB), inclusion and exclusion criteria application (SS and LB), data abstraction (SS and LB), and quality assessment (SS and LB) in duplicate consistent with standard practice. Data were abstracted directly from the full text of the study.

### 2.5. Data Items

Data collected were the number of patients on LZD alone and LZD + a serotonergic agent, number of patients on LZD + 1 serotonergic agent, number of patients on LZD + >1 serotonergic agent, incidence of ST between groups, number of serotonergic agents, and scoring tool used to diagnose ST. Intervention characteristics obtained were the definition of serotonergic agents, the definition of ST, patient data source(s), and funding sources. Any missing summary statistics from included studies were calculated using raw data.

### 2.6. Study Risk of Bias Assessment

To assess publication bias among the included studies, authors SS and LB performed a visual inspection of the funnel plot and Egger’s test. Both the funnel plot and Egger’s test were performed using the R meta package (Vienna, Austria) [15,16]. We also used the Newcastle–Ottawa Scale to evaluate the quality of the studies included [17]. The NOS allowed us to thoroughly scrutinize and assign scores to each study based on their predefined criteria. Each study was given one point for each criterion of the NOS that was met. Studies that scored 5 or more points were included in the study. A summary of these quality assessments can be found in Table A2. 

### 2.7. Effect Measures

The incidence of ST is reported as crude incidence rate in percentage or over 1000 patients when percentages were too small to be easily interpreted, using raw data from the included studies. All between-group comparisons are reported as odds ratios for all outcomes. In cases where raw, unadjusted data were available, we calculated the odds ratios directly from these data. For added rigor, adjusted odds ratios, when available, were collected directly from included articles.

### 2.8. Synthesis Methods

Data extracted from eligible studies were entered into a table for ease of reference and statistical analysis. Given the anticipation of ST outcomes being relatively rare and a low expected heterogeneity between studies, we performed a thorough assessment of heterogeneity. This evaluation was carried out using both Cochran’s Q test and the I^2^ statistic, with a value of less than 50% being indicative of relatively low/moderate heterogeneity and supporting the application of a fixed model [18,19]. It is noteworthy that we recognize the potential for bias in the I^2^ statistic, particularly in smaller meta-analyses [18,19].

In anticipation of a small number of events, the imprecision of the meta-analysis was assessed using the confidence intervals of the point estimates and calculation of the optimal information criteria [20]. The Peto method was used, and the Mantel–-Haenszel method was used for sensitivity analysis [21,22,23]. All statistical analyses were carried out using two software tools, Comprehensive Meta-Analysis version 3.0 (Englewood, NJ, USA) and R (Vienna, Austria) [15,16]. We would like to clarify that this article is based solely on previously conducted studies and does not contain any new studies with human participants or animals performed by any of the authors. Furthermore, please note that no external funding was received for the execution of this study.

### 2.9. Reporting Bias Assessment

Completion of the NOS and critical evaluation of the rationale for missing data in each study were undertaken to assess the risk of reporting bias among included studies [24].

### 2.10. Certainty Assessment

To assess the confidence in the body of evidence for each outcome, we used the *Grading of Recommendations, Assessment, Development, and Evaluations (GRADE) Handbook* guidance and GRADEpro software (2023; McMaster University and Evidence Prime; ON, Canada) [25,26]. 

## 3. Results

The initial phase of our systematic review cast a wide net, identifying a total of 496 studies through our database queries. After the necessary removal of duplicates, this number was refined to 119 records. Subsequently, we excluded case series and review articles that were not initially filtered. This left 26 studies, out of which 12 failed to meet our inclusion and exclusion criteria. 

**Figure 1:**
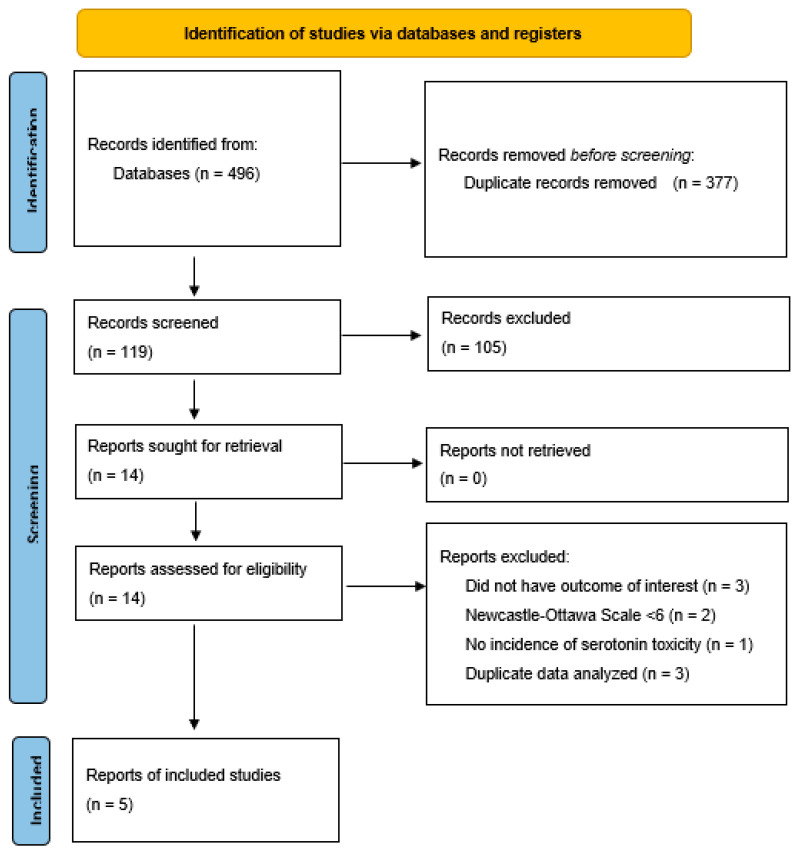
PRISMA^®^ 2020 flow diagram.

Fourteen full-text articles (consisting of nine observational studies and five case series) were then assessed for eligibility. Nine of these were excluded because of repetitive data (case series performed at different time points) [27,28,29], an inadequate NOS [5,6], missing outcomes of interest [9,30], or no ST in either group [8,31], leaving five studies in the final analysis [7,10,11,12,13]. Details of the selection process and key study characteristics can be found in Figure 1 and Table 1, respectively, providing readers with a transparent view of our research methodology and the study population. Furthermore, an in-depth evaluation of the quality of the included studies using the NOS is provided in Table A2. All included studies were retrospective cohort or retrospective case-control studies, as there were no RCTs analyzing the specified outcomes.

While five studies were included in the final analysis, not all studies could be used for each outcome. There were four studies and 6025 patients included in the comparison between LZD and LZD plus serotonergic agents [7,10,11,13]. Four studies and 2501 patients contained data for ST with LZD on one serotonergic agent versus >1 serotonergic agent and were used in this comparison [7,11,12,13]. Analyses were performed using the diagnosis of ST defined by the Hunter Serotonin Toxicity Criteria, except for one study (Thirot et al., 2018), as this study did not provide details of this assessment [13,32]. The Egger’s test did not identify any significant publication bias for either the comparison of ST incidence between LZD and LZD plus serotonergic agents or between LZD on one serotonergic agent versus > 1 serotonergic agent (*p* = 0.365 and 0.916, respectively). 

**Figure 2:**
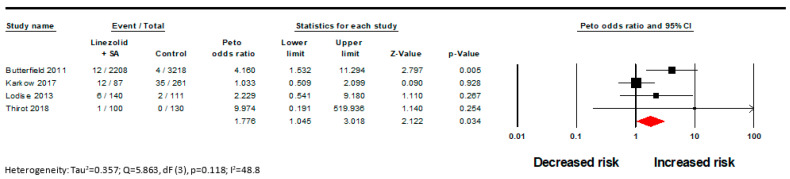
Forest plot examining the effect of linezolid + serotonergic agent versus linezolid monotherapy [7,10,11,13].

**Figure 3:**
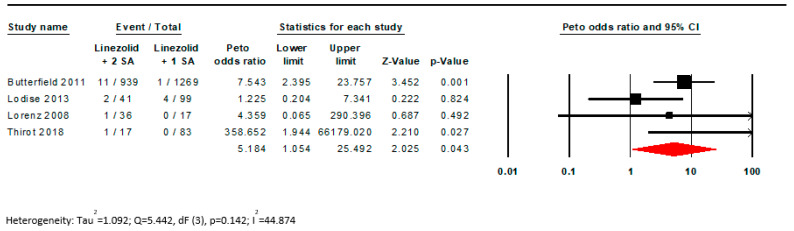
Forest plot examining the effect of linezolid + 1 serotonergic agonist versus linezolid + >1 serotonergic agents, demonstrating an increased risk with increasing serotonergic burden [7,11,12,13].

**Figure A1:**
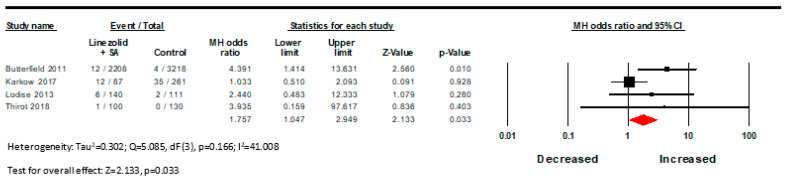
Forest plot examining the effect of linezolid + serotonergic agent versus linezolid monotherapy; sensitivity analysis using Mantel–Haenszel [7,10,11,13].

**Figure A2:**
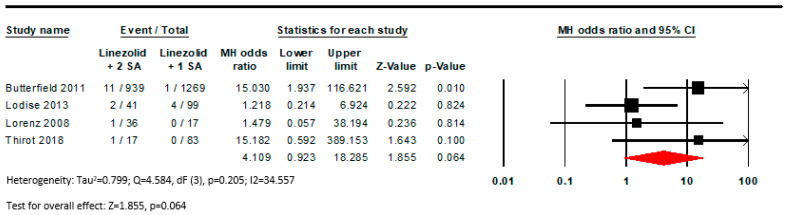
Forest plot examining the effect of linezolid + 1 serotonergic agent versus linezolid + >1 serotonergic agents; sensitivity analysis using Mantel–Haenszel [7,11,12,13].

There was a statistically significant difference found in the incidence of ST in LZD monotherapy versus LZD + a serotonergic agent with a low GRADE certainty assessment (OR 1.78; CI [1.04, 3.02]; I^2^ = 49%; Figure 2, Table 2). The pooled estimated incidence rate for this outcome was 12.3 per 1000 patients for those treated with LZD + a serotonergic agent versus 11 per 1000 patients treated with LZD monotherapy. Concomitant use of LZD with >1 serotonergic agent was associated with nearly a 5 times increased risk of ST versus LZD + a single serotonergic agent (OR 5.18; CI [1.05, 25.49]; I^2^ = 44.87; Figure 3). The GRADE certainty assessment for this outcome is moderate (Table 2) [25,26]. For this outcome, the pooled estimated incidence rate was 14.5 per 1000 patients for LZD + multiple serotonergic agents versus 3.4 per 1000 patients for LZD + 1 serotonergic agent. Similar results were seen in the Mantel–Haenszel model, although statistical significance was lost for the outcome of LZD with >1 serotonergic agent (OR 4.11; CI [0.92, 18.29]; I^2^ = 34.5; Figure A1 and Figure A2). 

In our sensitivity analysis, significance was lost for both outcomes with the removal of the Butterfield study, which extracted data from the locked databases of 20 Phase III and IV randomized control studies of LZD [7]. This study contained a large number of patients and likely influenced the outcome. 

## 4. Discussion

While our analyses revealed a statistically significant 1.77-fold increased risk of ST associated with the concomitant use of LZD and a serotonergic agent, it is essential to contextualize these findings within the broader perspective. Notably, this heightened risk, while statistically significant, must be interpreted in light of the inherently low incidence of ST events, affirming the rarity of this adverse event within the studied population. One must also consider that studies where no ST events occurred in either treatment group could not be included in our statistical analysis. A lack of inclusion of such double-zero studies will ultimately increase the numeric value of the estimated effect. Given that cases with no reported incidents of ST can be included in a meta-analysis, the observed results in our study must be understood as a likely overestimation of the association between LZD and serotonergic agents with ST. The outcomes of our analysis suggest that the actual association between LZD and serotonergic agents with ST may be lower than initially purported. Additionally, the relatively low magnitude of the effect size played a substantial role in our certainty assessment, leading to a decrease from moderate to low certainty regarding the strength of the association. 

We also report a significant positive association between multiple serotonergic agents with LZD versus a single serotonergic agent, a finding that has clinical implications. This finding suggests that the incidence of ST in patients receiving LZD is a function of overall serotonin burden. It is essential to approach this finding with a degree of caution, as the associated confidence interval is relatively wide. Nevertheless, this outcome adds to the body of evidence, pointing toward the relevance of considering the cumulative serotonin load when assessing the risk of ST in LZD-treated patients. Importantly, our moderate level of certainty in this evidence can be attributed, in part, to the robustness of the effect size, reinforcing the notion that the number of serotonergic agents administered alongside LZD may indeed hold greater clinical relevance than the presence of a single serotonergic agent. In the context of clinical decision-making, when evaluating the balance between the potential for iatrogenesis and the clinical benefits of treatment, it becomes imperative to weight the overall medication serotonin burden rather than focusing solely on individual drug–drug interactions. This holistic approach to risk assessment fosters a more comprehensive understanding of the factors contributing to ST and empowers clinicians with valuable insights for optimizing care. 

The results are not unexpected given the reversibility and degree of monoamine oxidase inhibitor (MAOI) activity by LZD. Early in vitro screening of LZD showed the inhibition of MAO-A, which led to preclinical animal and Phase I studies to evaluate the likelihood of significant MAO inhibition and drug interactions. These studies confirmed that linezolid is a relatively weak reversible competitive inhibitor of MAO-A (inhibition constant K_i_ = 55 µM) [33,34,35]. Linezolid has an observed C_max_ of 52.8 µM at steady state with 600 mg twice-daily dosing in healthy patients, which is similar to the K_i_ required for MAO-A interaction and could, under similar conditions (steady-state 600 mg twice daily), be of clinical relevance [3,35,36]. At doses higher than 600 mg twice daily or in situations where linezolid can accumulate, the C_max_ could exceed the K_i_ and lead to a drug interaction. 

This meta-analysis has some limitations. We acknowledge the limitations of not prospectively registering the protocol for the meta-analysis and the potential for such an omission to introduce reporting bias [14,24]. However, we believe we otherwise performed the study in accordance with the PRISMA Guidelines and have made the best effort to reduce bias in this meta-analysis. In addition, while we used three individual databases to identify relevant literature, it is possible that expanding our search to other databases may have yielded additional results. All studies included were retrospective and observational in nature. Individual studies did not report time to follow-up after drug administration. Most studies had a small sample size and had variations in the drugs considered serotonergic agents. Additionally, one study did not report the criteria they used to define ST, while all others used the Hunter Serotonin Toxicity Criteria (HSTC) or a modified version, which are both more sensitive and more specific than other commonly used criteria [32]. As the majority of studies did not adjust for confounding, there is potential that the odds ratio is influenced by unaccounted-for external variables. While the confidence interval for the primary meta-analysis was sufficiently narrow, there is a risk of bias in the degree of effect. Given the small number of outcomes, there is a risk of imprecision in the point estimates, which was the main influencing factor in the GRADE certainty assessment for both outcomes. As a further consideration, an optimal size criteria calculation was performed which was not met by the meta-analysis [20]. However, given the rarity of the outcome, the sample size for both outcomes was large. Regardless, the results affirm the risk of ST is rare. While larger prospective studies are necessary to definitively establish causation, it is unlikely that a large study will investigate this interaction due to the potential ethical concerns with treating patients using a drug combination that carries a potential risk of toxicity.

Certainly, building upon the context provided, the outcomes of this meta-analysis hold significant implications for clinical pharmacy practice. While acknowledging the increased risk of ST associated with the coadministration of LZD and serotonergic agents, it is crucial to recognize the rarity of ST events within the studied population. Therefore, in the clinical setting, a balanced approach is warranted. Clinicians must remain vigilant and consider the cumulative serotonin load when assessing patients receiving linezolid and serotonergic agents. In practice, this means tailoring medication regimens to the specific patient’s needs, risk factors, and treatment benefits while minimizing the unnecessary avoidance of potentially beneficial medications. In essence, these findings provide valuable insights for clinical pharmacists to optimize patient care, improve risk assessment, and support well-informed, patient-centered decisions in medication management.

## 5. Conclusions

These results suggest that linezolid could be used safely in patients on a single serotonergic agent if the benefits outweigh the risks evidenced by the low incidence of serotonin toxicity as a drug–drug interaction between linezolid and serotonergic agents. The overall serotonergic burden may be a clinically more relevant factor than simply the presence of serotonergic agents, as there was a 5 times higher rate of ST found in patients on multiple serotonergic agents + LZD than in patients on a single serotonergic agent + LZD. The results should be interpreted with caution given the relatively small number of outcomes and few studies included in the analysis.

## Figures and Tables

**Table 1:** Summary of included studies.

Study	Year	Country	Setting	Design	Definition of Exposure	Definition of Outcome	ST Rate, Cases per 1000 Patients (n/N)			
							LZD + SA	LZD + no SA	LZD + 1 SA	LZD + >1 SA
Butterfield et al. [7]	2011	United States	Phase III/IV RCTs	Retrospective cohort	Received any SA	Satisfy HSTC * or Sternbach criteria	5.4 (12/2208)	1.2 (4/3218)	0.8 (1/1269)	11.7 11/939
Karkow et al. [10]	2017	United States	Inpatients, University of Iowa Hospitals and Clinics	Retrospective matched cohort	Received LZD with or within 14 days of SA	Satisfy HSTC or Sternbach criteria	138 (12/87)	134 (35/261)	-	-
Lodise et al. [11]	2013	United States	Inpatients, VISN-2 ^†^	Retrospective matched cohort	At least 1 LZD dose + SA from −35 to + 7 days post treatment	Satisfy HSTC or Sternbach criteria	42.8 (6/140)	18 (2/111)	40 (4/99)	49 (2/41)
Lorenz et al. [12]	2008	United States	Inpatients, MUSC ^‡^	Retrospective cohort	SA concurrent or within 14 days of LZD	HSTC or surrogate signs/symptoms	18.9 (1/53)	-	0 (0/17)	27.8 (1/36)
Thirot et al. [13]	2018	Belgium	Inpatients,4 hospital centers	Retrospective cohort	LZD + SA	Not reported	10 (1/100)	0 (0/130)	0 (0/83)	58.8 (1/17)
Pooled incidence							12.3 (32/2588)	11 (41/3720)	3.4 (5/1468)	14.5 (15/1033)

* HSTC = Hunter Serotonin Toxicity Criteria; ^†^ VISN-2 = Veterans Integrated Service Network 2, Upstate New York Veterans Affairs Healthcare Network; ^‡^ MUSC = Medical University of South Carolina; LZD = linezolid; SA = serotonergic agent; ST = serotonin toxicity.

**Table 2:** GRADE evidence summary.

Certainty Assessment							# of Patients		Effect		Certainty
# of Studies	Study Design	Risk of Bias	Inconsistency	Indirectness	Imprecision	Other Considerations	[Intervention]	[Comparison]	Relative (95% CI)	Absolute (95% CI)	
**Serotonin Toxicity in LZD + SA versus LZD alone**											
4	Observational studies	Not serious	Not serious	Not serious	Serious ^a^	All plausible residual confounding would suggest spurious effect, while no effect was observed	31/2535 (1.2%)	41/3720 (1.1%)	**OR 1.750** (1.047 to 2.949)	**8 more per 1000** (from 1 more to 21 more)	⨁⨁◯◯ Low
**Serotonin Toxicity with LZD + 1 SA versus LZD + multiple SA**											
4	Observational studies	Not serious	Not serious	Not serious	Serious ^a^	Strong association all plausible residual confounding would suggest spurious effect, while no effect was observed	15/1015 (1.5%)	5/1486 (0.3%)	**OR 6.770** (2.240 to 20.447)	**19 more per 1000** (from 4 more to 61 more)	⨁⨁⨁◯ Moderate

CI: confidence interval; OR: odds ratio; LZD: linezolid; SA: serotonergic agent; Explanations: ^a^ Optimal information size was not met. Although the sample size is large given the rarity of the event, a larger sample would be ideal.

## Appendix A

**Table A1:** PRISMA 2009 Checklist.

Section and Topic	Item #	Checklist Item	Location Where Item Is Reported
**TITLE**			
Title	1	Identify the report as a systematic review.	Title
**ABSTRACT**			
Abstract	2	See the PRISMA 2020 for Abstracts checklist.	
**INTRODUCTION**			
Rationale	3	Describe the rationale for the review in the context of existing knowledge.	p. 3
Objectives	4	Provide an explicit statement of the objective(s) or question(s) the review addresses.	p. 3
**METHODS**			
Eligibility criteria	5	Specify the inclusion and exclusion criteria for the review and how studies were grouped for the syntheses.	p. 4
Information sources	6	Specify all databases, registers, websites, organisations, reference lists and other sources searched or consulted to identify studies. Specify the date when each source was last searched or consulted.	p. 4
Search strategy	7	Present the full search strategies for all databases, registers and websites, including any filters and limits used.	p. 4
Selection process	8	Specify the methods used to decide whether a study met the inclusion criteria of the review, including how many reviewers screened each record and each report retrieved, whether they worked independently, and if applicable, details of automation tools used in the process.	p. 5
Data collection process	9	Specify the methods used to collect data from reports, including how many reviewers collected data from each report, whether they worked independently, any processes for obtaining or confirming data from study investigators, and if applicable, details of automation tools used in the process.	p. 5
Data items	10a	List and define all outcomes for which data were sought. Specify whether all results that were compatible with each outcome domain in each study were sought (e.g., for all measures, time points, analyses), and if not, the methods used to decide which results to collect.	p. 4
	10b	List and define all other variables for which data were sought (e.g., participant and intervention characteristics, funding sources). Describe any assumptions made about any missing or unclear information.	p. 5
Study risk of bias assessment	11	Specify the methods used to assess risk of bias in the included studies, including details of the tool(s) used, how many reviewers assessed each study and whether they worked independently, and if applicable, details of automation tools used in the process.	p. 5
Effect measures	12	Specify for each outcome the effect measure(s) (e.g., risk ratio, mean difference) used in the synthesis or presentation of results.	p. 5
Synthesis methods	13a	Describe the processes used to decide which studies were eligible for each synthesis (e.g., tabulating the study intervention characteristics and comparing against the planned groups for each synthesis (item #5)).	p. 5
	13b	Describe any methods required to prepare the data for presentation or synthesis, such as handling of missing summary statistics, or data conversions.	p. 5
	13c	Describe any methods used to tabulate or visually display results of individual studies and syntheses.	Table 1
	13d	Describe any methods used to synthesize results and provide a rationale for the choice(s). If meta-analysis was performed, describe the model(s), method(s) to identify the presence and extent of statistical heterogeneity, and software package(s) used.	p. 5
	13e	Describe any methods used to explore possible causes of heterogeneity among study results (e.g., subgroup analysis, meta-regression).	p. 5
	13f	Describe any sensitivity analyses conducted to assess robustness of the synthesized results.	p. 5
Reporting bias assessment	14	Describe any methods used to assess risk of bias due to missing results in a synthesis (arising from reporting biases).	p. 5
Certainty assessment	15	Describe any methods used to assess certainty (or confidence) in the body of evidence for an outcome.	p. 5
**RESULTS**			
Study selection	16a	Describe the results of the search and selection process, from the number of records identified in the search to the number of studies included in the review, ideally using a flow diagram.	Figure 1
	16b	Cite studies that might appear to meet the inclusion criteria, but which were excluded, and explain why they were excluded.	p. 6
Study characteristics	17	Cite each included study and present its characteristics.	p. 6, Table 1
Risk of bias in studies	18	Present assessments of risk of bias for each included study.	Appendix A Table A2
Results of individual studies	19	For all outcomes, present, for each study: (a) summary statistics for each group (where appropriate) and (b) an effect estimate and its precision (e.g., confidence/credible interval), ideally using structured tables or plots.	Table 1
Results of syntheses	20a	For each synthesis, briefly summarise the characteristics and risk of bias among contributing studies.	p. 6, Table 1 and Table 2, Figure 2 and Figure 3
	20b	Present results of all statistical syntheses conducted. If meta-analysis was done, present for each the summary estimate and its precision (e.g., confidence/credible interval) and measures of statistical heterogeneity. If comparing groups, describe the direction of the effect.	p. 6, Figure 2 and Figure 3
	20c	Present results of all investigations of possible causes of heterogeneity among study results.	p. 6, Figure 2 and Figure 3
	20d	Present results of all sensitivity analyses conducted to assess the robustness of the synthesized results.	p. 6, Appendix A Figure A1 and Figure A2
Reporting biases	21	Present assessments of risk of bias due to missing results (arising from reporting biases) for each synthesis assessed.	n/a
Certainty of evidence	22	Present assessments of certainty (or confidence) in the body of evidence for each outcome assessed.	p. 6, p. 7
**DISCUSSION**			
Discussion	23a	Provide a general interpretation of the results in the context of other evidence.	p. 7, p. 8
	23b	Discuss any limitations of the evidence included in the review.	p. 7, p. 8
	23c	Discuss any limitations of the review processes used.	p. 7, p. 8
	23d	Discuss implications of the results for practice, policy, and future research.	p. 7, p. 8
**OTHER INFORMATION**			
Registration and protocol	24a	Provide registration information for the review, including register name and registration number, or state that the review was not registered.	p. 4
	24b	Indicate where the review protocol can be accessed, or state that a protocol was not prepared.	p. 4
	24c	Describe and explain any amendments to information provided at registration or in the protocol.	n/a
Support	25	Describe sources of financial or non-financial support for the review, and the role of the funders or sponsors in the review.	p. 5
Competing interests	26	Declare any competing interests of review authors.	p. 9
Availability of data, code and other materials	27	Report which of the following are publicly available and where they can be found: template data collection forms; data extracted from included studies; data used for all analyses; analytic code; any other materials used in the review.	n/a

From: Page MJ, McKenzie JE, Bossuyt PM, Boutron I, Hoffmann TC, Mulrow CD, et al. The PRISMA 2020 statement: an updated guideline for reporting systematic reviews. BMJ 2021;372:n71. doi: 10.1136/bmj.n71. [14]

**Table A2:** Newcastle–Ottawa scores.

	Domain	Butterfield 2011 [7]	Gatti 2020 [5]	Karkow 2017 [10]	Lodise 2013 [11]	Lorenz 2008 [12]	Quinn 2009 [6]	Thirot 2018 [13]
Selection	Representativeness/case definition	1	1	1	1	1	1	0
	Selection of nonexposed/cases	1	0	1	1	1	0	1
	Ascertainment of exposure/selection of controls	1	1	1	1	1	1	1
	Baseline assessment/definition of controls	1	1	1	1	1	1	1
Comparability	Confounders identified	1	0	1	1	1	0	1
	Statistical adjustment	0	0	0	1	0	1	1
Outcome/exposure	Outcome/exposure assessment	1	1	1	1	0	1	1
	Follow-up/method for ascertainment of exposure	1	1	1	1	1	1	1
	Adequacy of follow-up/nonresponse rate	1	0	1	1	1	0	1
	**Total Score**	**8**	**5**	**8**	**9**	**7**	**5**	**7**
